# Demographic and Clinical Insights Into Pyoderma Gangrenosum: A Retrospective Cross‐Sectional Study From a Tertiary Care Hospital

**DOI:** 10.1002/hsr2.72379

**Published:** 2026-04-16

**Authors:** Mitra Mirzaei, Alireza Jafarzadeh, Sepideh Salehi, Azadeh Goodarzi

**Affiliations:** ^1^ School of Medicine Iran University of Medical Sciences Tehran Iran; ^2^ Department of Dermatology, School of Medicine, Rasool Akram Medical Complex Clinical Research Development Center (RCRDC) Iran University of Medical Sciences Tehran Iran; ^3^ Department Of Dentistry Shahid Beheshti University Of Medical Sciences Tehran Iran

**Keywords:** differential diagnosis, inflammatory bowel disease, malignancy, pyoderma gangrenosum, systemic disorders, treatment satisfaction

## Abstract

**Background and Aims:**

Pyoderma gangrenosum (PG) is a rare, non‐infectious inflammatory skin disease often associated with systemic disorders such as inflammatory bowel disease (IBD), malignancies, and autoimmune conditions. This study aimed to investigate the demographic characteristics, comorbidities, differential diagnoses, and treatment outcomes in patients with histopathologically confirmed PG at a tertiary referral hospital in Iran.

**Methods:**

This retrospective cross‐sectional study included 58 patients diagnosed with PG at Rasool Akram Hospital between 2018 and 2023. Data were extracted from medical records and follow‐up phone interviews. Variables included demographic information, smoking status, pathergy test results, underlying diseases, treatment regimens, and patient‐reported satisfaction. Statistical analysis was performed using SPSS version 26.

**Results:**

Among the 58 patients, 53.4% were female, and the mean age was 55.88 ± 13.83 years. Underlying conditions were present in 46.6% of patients, most commonly IBD (29.3%), malignancy (15.5%), and rheumatoid arthritis (10.3%). The most prescribed medications were prednisolone (96.6%), methotrexate (51.7%), and cyclosporine (39.7%). Patient satisfaction was highest for etanercept (100%), infliximab (83.3%), and methotrexate (60.0%). No statistically significant associations were found between gender and smoking status or between comorbidities and pathergy test results.

**Conclusion:**

PG is frequently associated with systemic diseases, particularly IBD and malignancy, suggesting a syndromic relationship. Multidisciplinary evaluation is recommended for affected patients. Treatment satisfaction was highest for biologics and immunosuppressants, providing direction for future clinical management and research.

## Summary

- **What is already known about this topic?**
  Pyoderma gangrenosum (PG) is a non‐infectious inflammatory skin disease often linked with systemic conditions, particularly inflammatory bowel disease (IBD). It primarily affects individuals aged 30 to 55, with a higher occurrence in women, and is characterized by painful ulcers, most commonly on the lower legs.
  Treatment strategies depend on the severity of the lesions; mild cases are typically managed with local therapies, while more severe cases may necessitate the use of systemic corticosteroids or immunosuppressive agents.

- **What does this study add?**
  The study involved 58 patients with PG, predominantly females (53.4%) with an average age of 55.88 years. Nearly half (46.6%) had at least one underlying condition, most commonly IBD (29.3%), malignancies (15.5%), and rheumatoid arthritis (10.3%).
  A notable 43.1% of patients had a history of smoking, with 17.2% being current smokers. The average interval between quitting smoking and the diagnosis of PG was about 4.7 months, indicating the potential importance of smoking history in disease onset.
  Prednisolone was the most frequently used medication (96.6%), followed by methotrexate (51.7%) and cyclosporine (39.7%). Patient satisfaction was particularly high for etanercept (100% satisfaction), infliximab (83.3%), and methotrexate (60.0%), suggesting effective treatment options for managing PG.
  The high prevalence of associated conditions such as IBD and malignancies indicates a potential syndromic relationship. This underscores the necessity for multidisciplinary referrals to assess and manage co‐occurring health issues in patients with PG.

## 1 Introduction

Pyoderma gangrenosum (PG) is a non‐infectious, neutrophilic, and ulcerative inflammatory skin disease frequently associated with systemic conditions [1, 2]. Initially described by Brook in 1916, PG's prevalence in inflammatory bowel disease (IBD) was later studied by Krenitsky et al. in 1976 [1]. It can affect individuals of any age but is most common between 30 and 55 years, with a slightly earlier onset in women than men [3, 4]. Lesions typically appear on the lower legs, and the disease is characterized by an uncertain etiology and complex pathophysiology, believed to be multifactorial, involving immune dysregulation, inflammatory cytokines, neutrophil activation, and genetic factors [5, 6].

Clinically, PG presents in several types: the Classic or Ulcerative type appears as painful pustules that evolve into ulcers with purplish borders; the Pustular type occurs during IBD flare‐ups; the Bullous or Atypical type features superficial hemorrhagic blisters linked to myeloproliferative disorders; and the Vegetative type is marked by solitary, painless ulcers without purplish margins [3].

Diagnosis primarily relies on clinical evaluation and exclusion of other ulcerative causes, given its non‐specific pathological representation [7]. Differential diagnoses include Wegener's granulomatosis, Antiphospholipid syndrome, Behçet's disease, and others [8]. Approximately 50%–70% of PG cases are associated with systemic disorders, most commonly IBD, along with leukemia, myeloma, and various autoimmune conditions [1, 9].

Treatment varies based on lesion type and underlying diseases. Mild cases may respond to local treatments, such as wet compresses, hydrophilic dressings, and topical steroids, while more severe cases often require systemic corticosteroids or additional immunosuppressive agents [9]. Early diagnosis and corticosteroid initiation are crucial for effective management, especially in peristomal PG cases [10].

Despite numerous studies on the demographics, associated disorders, differential diagnoses, and treatment options of PG, there remain significant gaps in research—particularly from tertiary care hospitals in the Middle East. Given the diagnostic complexity and frequent syndromic associations of PG, comprehensive data from advanced clinical settings are essential to improving diagnostic accuracy and clinical decision‐making. This retrospective cross‐sectional study aims to address this gap by analyzing the demographic features, comorbid conditions, and treatment patterns of patients with histopathologically confirmed PG over an 18‐year period at a major referral center. The findings may enhance early recognition, guide interdisciplinary referrals, and support evidence‐based management strategies for high‐risk patient populations.

## 2 Methods

### 2.1 Study Design and Setting

This was a retrospective cross‐sectional study conducted at Rasool Akram Hospital, a major tertiary referral center affiliated with Iran University of Medical Sciences (IUMS) in Tehran, Iran. The study was conducted in accordance with the STROBE (Strengthening the Reporting of Observational Studies in Epidemiology) checklist.

### 2.2 Participants

The study included all patients with a histopathologically confirmed diagnosis of PG referred to Rasool Akram Hospital between 2018 and 2023. No sampling was performed, as the entire available population was reviewed. The inclusion criterion was confirmed PG diagnosis; the only exclusion criterion was refusal to participate.

### 2.3 Diagnostic Framework

In this study, the diagnosis of PG was primarily based on clinical evaluation and histopathological confirmation. To enhance diagnostic accuracy and standardize the process, the PARACELSUS score [8] was used as a structured diagnostic tool. This score is based on three major criteria and several minor criteria to assess the severity and confirm the diagnosis of PG.

- Major Criteria:
  1. Rapid disease progression
  2. Assessment of relevant differential diagnoses
  3. Reddish‐violaceous wound borders

- Minor Criteria:
  1. Improvement with immunosuppressive drugs
  2. Irregular ulcer shape
  3. Severe pain (≥ 4/10 on the visual analog scale)
  4. Lesion location at the site of trauma

All patients with histopathologically confirmed PG were evaluated based on the PARACELSUS score. In cases where clinical and histopathological diagnoses were ambiguous, a final diagnosis was made by consensus between two experienced dermatologists.

### 2.4 Scoring Standards

Scoring was performed based on a combination of the major and minor criteria. In this study, a diagnostic threshold was set, so only patients who scored above a predetermined threshold were considered to have a definitive diagnosis of PG. This approach aimed to improve diagnostic precision and clarity in subsequent treatment decisions.

### 2.5 Patient Satisfaction Measurement

Patient satisfaction in this study was measured using a 5‐point Likert scale, ranging from “completely dissatisfied” to “completely satisfied.” The scale was as follows:

- Completely dissatisfied;
- Dissatisfied;
- Neutral;
- Satisfied;
- Completely satisfied.

Satisfaction was assessed 6 weeks after the initiation of treatment and during follow‐up visits. Therefore, patient satisfaction was measured at one time point (6 weeks post‐treatment). The satisfaction assessment was conducted using a standardized questionnaire specifically designed to evaluate satisfaction with dermatological treatments.

Data were collected through telephone interviews and online questionnaires. These questionnaires were sent directly to the patients after treatment, and where necessary, telephone interviews were conducted to complement the responses.

### 2.6 Validation of the Satisfaction Tool for PG

The tool used to measure patient satisfaction has been validated in previous studies related to dermatological treatments, including PG. This scale has been widely accepted as a reliable measure of satisfaction in skin disease treatment studies, ensuring its relevance and accuracy for PG‐related treatments [11, 12].

### 2.7 Handling of Missing Data

To handle missing data, the Missing Completely at Random (MCAR) approach was used. This means that the missing data were assumed to be random and unrelated to other variables, and therefore, missing data were excluded from the analysis. If sensitivity analyses are required to evaluate the impact of missing data, the results will be discussed in Section 4.

### 2.8 Data Collection

Data were extracted from clinical records and telephone interviews using a researcher‐designed checklist. Collected variables included age, gender, smoking status, comorbidities (e.g., IBD, malignancy, RA), pathergy test results, medications used, treatment satisfaction, and initial differential diagnoses.

### 2.9 Ethical Considerations

This study was approved by the Ethics Committee of the Iran University of Medical Sciences (IR.IUMS.REC.1399.1776). All procedures were conducted in accordance with the ethical standards of the institutional and national research committee and with the 1964 Helsinki declaration and its later amendments. Verbal informed consent was obtained from all participants after the aims and methodology of the study were clearly explained to them via telephone, and their participation was entirely voluntary. All patient data were anonymized to ensure confidentiality.

### 2.10 Statistical Analysis

All analyses were conducted using IBM SPSS Statistics for Windows, Version 26.0 (IBM Corp., Armonk, NY, USA). Statistical methods included the Kolmogorov–Smirnov test to assess normality, Chi‐square tests for evaluating associations between categorical variables, and Fisher's exact test when expected frequencies were small. All statistical tests were two‐sided, and a *p*‐value of less than 0.05 was considered statistically significant. The primary analyses, including associations between treatment types and satisfaction, were pre‐specified. Subgroup analyses based on comorbidities were exploratory and descriptive due to small subgroup sizes. Statistical terms, abbreviations, and symbols are defined where first used in the manuscript. These analyses were performed in accordance with the SAMPL (Statistical Analyses and Methods in the Published Literature) guidelines.

## 3 Results

In this study, we examined 58 patients diagnosed with PG who were referred to the Hospital.

### 3.1 Demographic and Clinical Characteristics

Regarding gender distribution, there were 31 female patients (53.4%) and 27 male patients (46.6%). The average age of the participants was 55.88 ± 13.83 years, with ages ranging from 19 to 83 years.

### 3.2 Smoking Status and Pathergy Test Results

**Figure 1:**
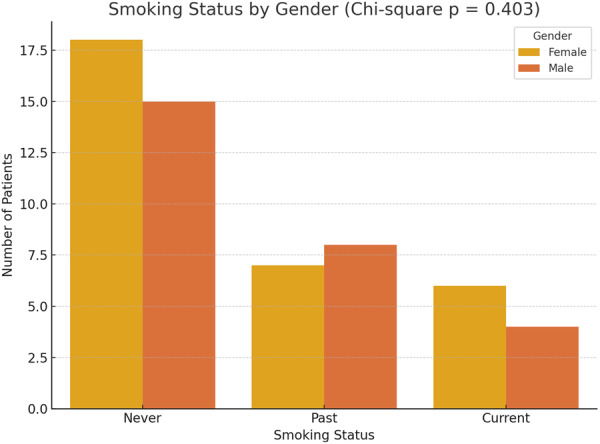
Bar chart showing smoking status distribution by gender. No statistically significant association was found (Chi‐square *p* = 0.403) despite visual variation.

**Figure 2:**
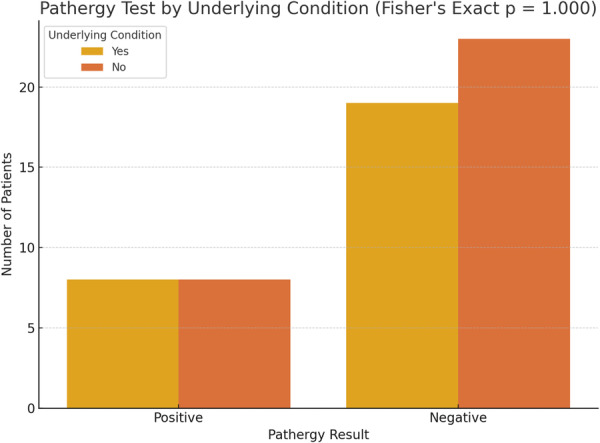
Bar chart displaying pathergy test results in patients with and without underlying conditions. No significant association was found (Fisher's Exact Test *p* = 1.000).

Regarding smoking history, 10 patients (17.2%) were current smokers, while 15 patients (25.9%) reported a past history of smoking. Among the former smokers, the average time interval between smoking cessation and the diagnosis of PG was 4.7 ± 1.3 months. Additionally, the pathergy test was positive in 16 patients (27.6%). To assess potential associations, a Chi‐square test was conducted to examine the relationship between gender and smoking status (categorized as never, past, or current smokers). The analysis revealed no statistically significant association between gender and smoking status (Chi‐square test, *p* = 0.403). Likewise, no significant association was observed between gender and pathergy test positivity (*p* = 1.000) (Figures 1 and 2).

### 3.3 Underlying Conditions

The prevalence of underlying systemic diseases was assessed, revealing that 27 patients (46.6%) had at least one comorbid condition. The most common underlying diseases were IBD in 17 patients (29.3%), malignancy in 9 patients (15.5%), rheumatoid arthritis in 6 patients (10.3%), and gammopathy in 4 patients (6.9%).

Among the patients with IBD, nine were diagnosed with Crohn's disease and eight with ulcerative colitis. Of the nine patients with malignancies, three had myelodysplastic syndromes, two had acute leukemia, and one patient each had ovarian cancer, chronic leukemia, brain cancer, and Hodgkin lymphoma. To evaluate whether the presence of an underlying systemic condition (such as IBD, malignancy, rheumatoid arthritis, or gammopathy) was associated with pathergy test positivity, Fisher's Exact Test was performed. The result indicated no statistically significant association between the presence of an underlying disease and a positive pathergy test (Fisher's Exact Test, *p* > 0.99).

### 3.4 Medication Usage

**Figure 3:**
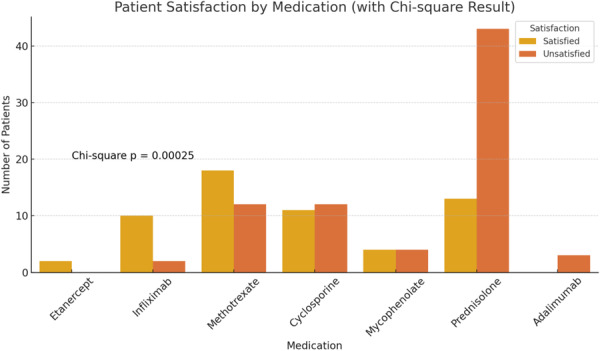
Stacked bar plot of patient satisfaction by medication. Statistically significant differences observed across drugs (Chi‐square *p* = 0.00025).

An analysis of the patients' medication usage revealed that the most commonly prescribed treatments were prednisolone in 56 cases (96.6%), methotrexate in 30 cases (51.7%), and cyclosporine in 23 cases (39.7%) (Table 1). To determine whether the level of patient satisfaction varied significantly across different medications, a Chi‐square test was performed. The Chi‐square test (*χ*² = 25.69, df = 6) showed a statistically significant difference in patient satisfaction across medications (*p* < 0.001). This indicates a statistically significant association between the type of medication used and patient satisfaction. Specifically, certain drugs, such as etanercept and infliximab, were associated with notably higher satisfaction rates compared to others like prednisolone and adalimumab (Figure 3).

**Table 1:** Frequency of medications administered to patients.

Drug name	Frequency	Percentage
Prednisolone	56	96.6
Methotrexate	30	51.7
cyclosporine	23	39.7
Infliximab	12	20.7
Mycophenolate mofetil	8	13.8
Adalimumab	3	5.2
Etanercept	2	3.4

### 3.5 Treatment Satisfaction

Patient feedback on treatment satisfaction, based on medication use, indicated varying levels of satisfaction across different drugs. Etanercept had the highest satisfaction rate, with two out of two patients (100.0%) expressing satisfaction (Grades 4 and 5), followed by infliximab with 10 out of 12 patients (83.3%), and methotrexate with 18 out of 30 patients (60.0%) (Table 2).

**Table 2:** Patient satisfaction levels regarding treatment based on the medication utilized.

Drug name	Frequency of use	Frequency of patients' satisfaction	Percentage of patients' satisfaction
Prednisolone	56	13	23.2
Methotrexate	30	18	60.0
cyclosporine	23	11	47.8
Infliximab	12	10	83.3
Mycophenolate mofetil	8	4	50.0
Adalimumab	3	0	0.0
Etanercept	2	2	100.0

When treatment satisfaction (Grades 4 and 5) was analyzed in relation to comorbidities, prednisolone was the most commonly prescribed medication across all groups. However, the highest satisfaction rates varied by condition: for patients with IBD, infliximab yielded the highest satisfaction (83.3%); for those with malignancy, methotrexate was most effective (63.2%); for rheumatoid arthritis, etanercept achieved full satisfaction (100.0%); and for gammopathy, methotrexate again showed the highest satisfaction (75.0%) (Table 3).

**Table 3:** Patient satisfaction with treatment based on comorbidities.

Medical history	Frequency	The highest rate of treatment satisfaction
Inflammatory bowel disease	17	Infliximab (83.3%)
Malignancy	9	Methotrexate (63.2%)
Rheumatoid arthritis	6	Etanercept (100.0%)
Gammopathy	4	Methotrexate (75.0%)

Due to the small number of patients in each comorbidity subgroup (e.g., only four patients with gammopathy), formal statistical testing such as Chi‐square or Fisher's exact test was not feasible. As a result, these findings are presented descriptively. Nevertheless, the descriptive data suggest a potential trend in treatment effectiveness linked to specific comorbidities.

### 3.6 Differential Diagnoses

An assessment of the initial differential diagnoses proposed for patients revealed that the most commonly suspected conditions were vascular obstructive diseases (17 cases, 29.3%), vasculitides (13 cases, 22.4%), malignancies (13 cases, 22.4%), and infectious diseases (9 cases, 15.5%) (Table 4).

**Table 4:** Frequency of initial differential diagnoses proposed for patients.

Differential diagnosis	Frequency	Percentage
Vascular diseases	17	29.3
Vasculitis	13	22.4
Malignancies	13	22.4
Infectious diseases	9	15.5
Drug Reactions	2	3.4
Other diagnosis	4	6.9

**Figure 4:**
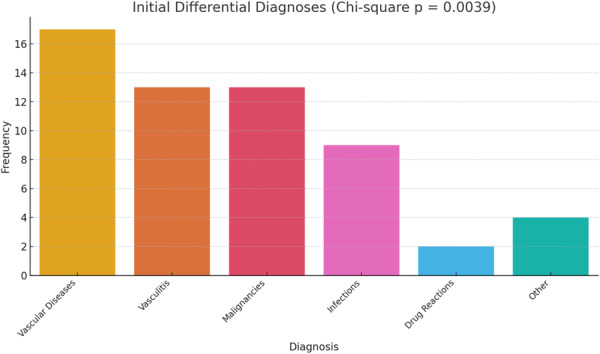
Bar chart showing the distribution of initial differential diagnoses. A statistically significant difference was observed across categories (Chi‐square *p* = 0.0039), indicating clinicians more frequently suspected conditions like vascular diseases and vasculitis.

To evaluate whether this distribution differed significantly from a uniform distribution, a Chi‐square goodness‐of‐fit test was conducted. A Chi‐square test (*χ*² = 17.31) indicated a statistically significant non‐uniform distribution of initial differential diagnoses (*p* = 0.004). This suggests that clinicians were more likely to initially suspect certain conditions, such as vascular diseases and vasculitis, over less common alternatives like drug reactions or rare disorders (Figure 4).

## 4 Discussion

This study analyzed 58 patients diagnosed with PG over a 5‐year period at a tertiary care referral hospital. The observed female predominance (53.4%) and average age of 55.88 years are consistent with earlier international data, supporting the reliability of the findings.

In the study conducted by Caldarola et al. [13], 35 individuals with PG were examined, consisting of 22 women and 13 men, with an average age of 40 years. This finding aligns with our study, which also showed a female predominance among the patients. Among the participants, 57.1% had multiple affected areas, with the lower limbs being the most frequently involved (85.7%). PG was associated with IBD in 51.4% of cases and with hidradenitis suppurativa in 37.1%. Biologic treatments led to better outcomes, with 82% of treated patients achieving full skin re‐epithelialization. The study highlighted that biologic drugs, particularly infliximab, were more effective in promoting faster and more complete skin healing compared to traditional therapies. These findings support our own results.

Our findings align with Vacas et al. [4], who reported IBD in approximately 32% of PG patients and emphasized the syndromic relationship of PG with systemic diseases. Similarly, a large cohort study by Jockenhöfer et al. [8] confirmed that nearly half of PG patients had at least one autoimmune or malignant comorbidity. These correlations further support the importance of a multidisciplinary approach to PG management.

In a systematic review conducted by Tan et al. [14], 107 studies encompassing 139 patients with PG were evaluated. Among these cases, 92 patients received TNF‐α inhibitors, whereas 47 were treated with interleukin (IL) inhibitors. Infliximab and adalimumab were the most frequently prescribed TNF‐α inhibitors and showed favorable efficacy outcomes, while IL inhibitors exhibited similar or superior therapeutic effectiveness. Complete remission was achieved in 46.0% of patients, and combined complete or partial remission rates reached 93.6% in the IL inhibitor group and 88.0% in the TNF‐α inhibitor group, with no statistically significant difference between treatment categories. However, IL inhibitors were associated with a shorter duration to clinical response. Conversely, infliximab was linked to a higher incidence and greater diversity of adverse events compared with other biologic therapies.

In a narrative review by Molinelli et al. [15], anti–TNF‐α therapies—most notably infliximab and adalimumab—were identified as the most commonly employed biologic agents for PG and were associated with the highest clinical response rates, particularly in refractory and moderate‐to‐severe disease. Infliximab demonstrated robust effectiveness across randomized controlled trials, retrospective analyses, and case series, with reported response rates ranging from 69% to 100% and rapid ulcer improvement frequently occurring within a few weeks. Similarly, adalimumab yielded favorable therapeutic outcomes, with complete ulcer resolution observed in over half of patients in open‐label studies and retrospective cohorts. Moreover, accumulating evidence from case reports and small case series suggests that non–TNF‐targeted biologics—including IL‐1, IL‐17, IL‐23, and IL‐36 inhibitors—as well as JAK inhibitors, may offer effective alternatives in multirefractory cases. Nevertheless, the authors noted that the current evidence base is largely derived from small‐scale and anecdotal studies, underscoring the need for well‐designed controlled trials to more clearly establish efficacy and safety.

A key novel contribution of this study is the evaluation of patient satisfaction across different treatment modalities. Biologic agents, particularly infliximab and etanercept, demonstrated the highest levels of patient satisfaction, underscoring their real‐world effectiveness. Consistent with our findings, the study by Alavi et al. [5] reported successful outcomes with infliximab in refractory cases of PG.

Although the associations between comorbidities and treatment responses were not statistically significant in our subgroup analysis (likely due to small sample sizes), the observed trends warrant further exploration. Kridin (2018) emphasized the heterogeneity of PG and the need for individualized therapy guided by comorbidity profiles and disease severity [3].

The diagnostic overlap between PG and other ulcerative conditions remains a significant clinical challenge. In our study, common initial differential diagnoses included vasculitis, vascular disorders, and malignancy, findings that are consistent with recent updates from the British Association of Dermatologists [16]. The non‐specific nature of histopathological features in PG underscores the need for careful exclusion of mimicking conditions through biopsy and close clinicopathological correlation.

Our treatment data suggest that corticosteroids remain the cornerstone of initial management, consistent with international guidelines. However, satisfaction rates were significantly higher for biologics, suggesting their superior long‐term outcomes. Jockenhöfer et al. [8] found that patients on infliximab had faster healing times and better quality of life outcomes than those treated with traditional immunosuppressants.

Compared with prior reports, this is one of the few regional studies from the Middle East to provide detailed insight into PG treatment satisfaction linked to systemic diseases. It underscores the need for multidisciplinary evaluation and treatment planning in PG patients. Furthermore, the structured analysis following STROBE guidelines enhances transparency and replicability, supporting this study's potential inclusion in future systematic reviews.

In conclusion, the study not only reaffirms the known associations of PG with IBD and malignancy but also provides new data on treatment satisfaction patterns. These findings can assist dermatologists and internists in early recognition, comorbidity screening, and optimizing therapeutic approaches. Future multicenter prospective studies with larger sample sizes are warranted to validate these findings and refine treatment algorithms for PG in diverse clinical settings.

## 5 Conclusion

The high prevalence of co‐occurring conditions such as IBD, malignancy, and rheumatoid arthritis suggests a possible syndromic relationship between these diseases. This highlights the importance of referring patients with PG to specialists in other fields for evaluation of potential comorbidities. Furthermore, the significant satisfaction reported from the use of etanercept, infliximab, and methotrexate, along with insights into the disease's pathophysiology, can guide future clinical trials and inform the selection of the most appropriate treatment strategies for patients.

## 6 Limitations

The process of reviewing medical records and documenting information was time‐intensive. Some existing records were either incomplete or difficult to read, which may have affected the comprehensiveness of our data. Additionally, a few patients were uncooperative regarding their participation in the study.

## Author Contributions

**Alireza Jafarzadeh:** methodology, formal analysis. **Azadeh Goodarzi:** conceptualization, supervision. All authors have read and approved the final version of the manuscript. The corresponding author, Dr. Azadeh Goodarzi, had full access to all of the data in this study and takes complete responsibility for the integrity of the data and the accuracy of the data analysis.

## Funding

This study did not receive any financial support or funding from public, commercial, or not‐for‐profit organizations. No funding body was involved in the design of the study; the collection, analysis, or interpretation of data; the writing of the manuscript; or the decision to submit the manuscript for publication.

## Ethics Statement

This study was approved by the Ethics Committee of the Iran University of Medical Sciences (IR.IUMS.REC.1399.1776). All procedures were conducted in accordance with the ethical standards of the institutional and national research committee and with the 1964 Helsinki declaration and its later amendments. Verbal informed consent was obtained from all participants after the aims and methodology of the study were clearly explained to them via telephone, and their participation was entirely voluntary. All patient data were anonymized to ensure confidentiality.

## Conflicts of Interest

The authors declare no conflicts of interest.

## Transparency Statement

The lead author, Azadeh Goodarzi, affirms that this manuscript is an honest, accurate, and transparent account of the study being reported; that no important aspects of the study have been omitted; and that any discrepancies from the study as planned (and, if relevant, registered) have been explained.

## Data Availability

The authors confirm that the data supporting the findings of this study are available within the article. Additional de‐identified data may be made available from the corresponding author, Dr. Azadeh Goodarzi, upon reasonable request.
